# CASE STUDY EXEMPLAR OF DETECTING SEVERE DIASTOLIC DYSFUNCTION USING BALLISTOCARDIOGRAM

**DOI:** 10.1093/geroni/igz038.339

**Published:** 2019-11-08

**Authors:** Laurel A Despins, Giovanna Guidoboni, Marjorie Skubic, Lorenzo Sala, Moein Enayati, James Keller, Mihail Popescu

**Affiliations:** 1 University of Missouri, Columbia, Missouri, United States; 2 Université de Strasbourg, Strasbourg Cedex, France

## Abstract

The specific aim of this case study was to describe how monitoring ballistocardiogram (BCG) waveforms can detect early heart failure (HF) changes. HF significantly impairs quality of life and is the principal cause for hospital readmissions in older adults. HF prevalence in American adults aged 65 years and older is expected to increase over 70% by 2030. Detecting worsening HF is challenging. Invasive arterial waveforms display blood pressure changes with each heartbeat; BCG waveforms display repetitive body motions resulting from ejection of blood into the great vessels. BCG waveforms change as cardiac function changes. Currently, BCG signals can be captured non-invasively using sensors placed under a bed mattress and provide heart and respiratory rates. We have developed a new way to analyze the BCG waveform using an innovative closed-loop physiological model of the cardiovascular system. The subject, a 94-year old female with hypertension, presented to her physician with symptoms associated with a new diagnosis of acute mixed congestive HF. Mean heart and respiratory rate trends obtained from her bed sensor in the prior two months did not indicate HF. We simulated cardiac cycles using normal cardiac function data, mildly impaired diastolic function data, and the subject’s echocardiography data. The results demonstrated BCG waveform changes that correlated with decreasing cardiac output related to worsening diastolic function. New methods for clinically interpreting BCG waveforms present a significant opportunity for improving early HF detection and improving outcomes. Working on a clinical problem from an engineering perspective merges two disciplines, creating a new methodology.

